# shinyCurves, a shiny web application to analyse multisource qPCR amplification data: a COVID-19 case study

**DOI:** 10.1186/s12859-021-04392-1

**Published:** 2021-10-03

**Authors:** S. Olaechea-Lázaro, I. García-Santisteban, J. R. Pineda, I. Badiola, S. Alonso, Jose Ramon Bilbao, Nora Fernandez-Jimenez

**Affiliations:** 1 Department of Genetics, Physical Anthropology and Animal Physiology, Faculty of Medicine and Nursing, University of the Basque Country (UPV/EHU), Barrio Sarriena s/n, 48940 Leioa, Spain; 2 Biocruces-Bizkaia Health Research Institute, Plaza de Cruces, 48903 Barakaldo, Spain; 3 Achucarro Basque Center for Neuroscience, Barrio Sarriena s/n, 48940 Leioa, Spain; 4 Department of Cell Biology and Histology, Faculty of Medicine and Nursing, University of the Basque Country (UPV/EHU), Barrio Sarriena s/n, 48940 Leioa, Spain

**Keywords:** Diagnosis, qRT-PCR, Melting and amplification curves, COVID-19, Data analysis, Medical informatics, Virology, Shiny application

## Abstract

**Background:**

Quantitative, reverse transcription PCR (qRT-PCR) is currently the gold-standard for SARS-CoV-2 detection and it is also used for detection of other virus. Manual data analysis of a small number of qRT-PCR plates per day is a relatively simple task, but automated, integrative strategies are needed if a laboratory is dealing with hundreds of plates per day, as is being the case in the COVID-19 pandemic.

**Results:**

Here we present shinyCurves, an online shiny-based, free software to analyze qRT-PCR amplification data from multi-plate and multi-platform formats. Our shiny application does not require any programming experience and is able to call samples Positive, Negative or Undetermined for viral infection according to a number of user-defined settings, apart from providing a complete set of melting and amplification curve plots for the visual inspection of results.

**Conclusions:**

shinyCurves is a flexible, integrative and user-friendly software that speeds-up the analysis of massive qRT-PCR data from different sources, with the possibility of automatically producing and evaluating melting and amplification curve plots.

**Supplementary Information:**

The online version contains supplementary material available at 10.1186/s12859-021-04392-1.

## Background

Quantitative, reverse transcription polymerase chain reaction (qRT-PCR) is a widely used technique for the detection and quantification of mRNA. Currently, with the spread of COVID-19, and despite the development of a number of interesting imaging and artificial intelligence-based diagnostic procedures [1, 2], qRT-PCR is still considered the gold-standard tool for SARS-CoV-2 detection by the World Health Organization (WHO) [3]. Depending on the amount of viral mRNA detected by the qRT-PCR system, a sample is assigned as Positive, Negative or Undetermined. Most healthcare providers rely on automated detection procedures that make use of proprietary reagents and software and offer limited options for parameter set-up, with special emphasis on those recommended by WHO (CFX Manager 3.1 from BioRad) and by the FDA (Food and Drug Administration) (the SDS 1.4 software from Applied Biosystems).

In this context, research laboratories worldwide are developing alternative diagnostic protocols that do not depend on commercial kits and their accompanying software. Thus, researchers may choose from a wide variety of quantitative gene expression reagents (one- vs. two-step qRT-PCR or fluorescent probes *vs*. intercalating dyes) and adapt protocols to the real-time amplification systems available [4]. In addition to the need for independent experimental protocols, there is also a need for an open-source tool that can rapidly analyze qRT-PCR data irrespective of the protocol or equipment used. Additionally, in order to discard samples with unspecific amplification products, a software for accurate qRT-PCR analysis should allow the inspection of amplification and melting curves.

Finally, the increase in the number of worldwide infections, driven by fast-spreading variants that are especially relevant in low-income countries, makes qRT-PCR the first choice for SARS-Cov-2 detection. There is a need for open-source tools for qRT-PCR data analysis that are easily customizable to the experimental set-up in each setting, without the prohibitive cost of proprietary licenses. Furthermore, the high risk in dense populations together with the close contact of SARS-CoV-2 reservoir in host’s animals makes the qRT-PCR detection an urgent need with strong public health implications [5, 6].

In this context, and taking into account both WHO recommendations and the new challenges of the global COVID-19 pandemic, the objective of our work was to develop a flexible, fast and non-proprietary software for massive qRT-PCR data analysis. Herein, we present shinyCurves, a Shiny-based, user-friendly, flexible, integrative, non-proprietary and free application that is able to:

1. Process qRT-PCR raw amplification data obtained with either fluorescent probes or intercalating dyes, from different plate formats and qRT-PCR systems, including those recommended by the main public health agencies;
2. Establish the settings that will classify samples into three categories (Positive, Negative or Undetermined), and to include both a range of optional experimental controls, and the possibility of using serial dilutions of viral RNA/DNA;
3. Plot both amplification and melting curves, providing additional quality control of the specificity of the amplification and offering the possibility of visually inspecting the results obtained.

A COVID-19 toy dataset is also provided as a practical example (see Additional file 1).

## Implementation

shinyCurves is designed for users with limited or no programming experience who wish to analyze qRT-PCR data in a simple and efficient manner. The application was completely written using R [7] in combination with the ‘shiny’ package [8], and can be found in the shinyapps.io repository as a web application (https://biosol.shinyapps.io/shinycurves/). The source code can be freely downloaded from the GitHub repository https://github.com/biosol/shinyCurves. Analysis tables are processed by ‘data.table’ [9] and ‘dplyr’ [10] due to their high efficiency, and plots are generated using ‘ggplot2’ [11] and ‘plotly‘ [12]. Specifically, to plot melting curves the ‘qpcR’ R package [13] is used.

**Figure 1.**
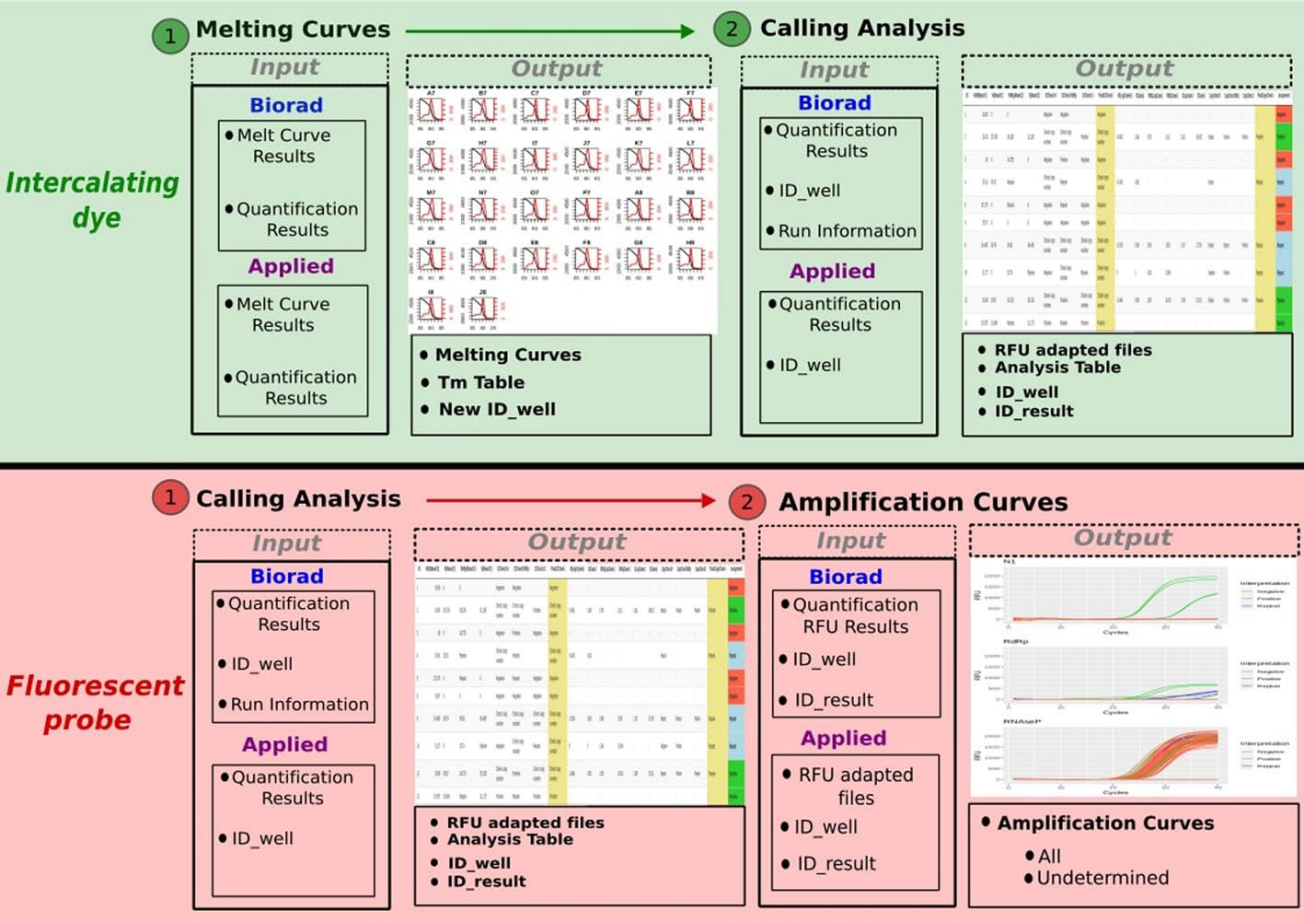
An overview example of two shinyCurves analyses (intercalating dye and fluorescent probe, in green and pink, respectively). Both analyses can be performed using qRT-PCR results from BioRad CFX or Applied Biosystems Quant Studio platforms, among others. The input data required for each analysis are specified in the black boxes. In green, the intercalating dye pipeline starts with the plotting of melting curves to discard unspecific amplification products. Then, in the calling analysis each sample is assigned a final result (Positive, Negative, Undetermined). In pink, the fluorescent probe pipeline starts with the calling analysis and is followed by the plotting of amplification curves for the visual inspection of the results

Raw data can be uploaded directly to the application in different file formats, including csv, xlsx or xls files generated in the two most widely used qPCR systems (i.e. BioRad and Applied Biosystems platforms), as pointed out in Fig. 1. Once the upload is complete, the intercalating dye pipeline will start by plotting a melting curve per reaction, in order to discard those that show unspecific amplification products, before sample classification into the Positive, Negative and Undetermined groups. In the case of the fluorescent probe pipeline, it will first perform the classification of the samples or the calling analysis, and then plot the amplification curves for visual inspection of the results.

Finally, it is worth to mention that shinyCurves allows for the use of sample duplicates and is also independent from the plate format, i.e. 96- or 364-well plates. The inclusion of experimental controls and serial dilutions of viral DNA is optional, but if included, must follow specific formats, as described in the provided Manual (see Additional file 2).

## Results

To illustrate the shinyCurves pipeline, data sets generated by the COVID-19 Basque Inter-Institutional Group (coBIG) in both fluorescent probe and intercalating dye experiments, and run on the BioRad CFX and Applied Biosystems Quant Studio systems are provided [4]. The genes chosen for these analyses are *N1*, *RdRp* and *RNAseP* (human genomic control) in the fluorescent probe assay, and *N*, *S*, *RdRp* and *H30* (endogenous control) in the intercalating dye assay. These genes are included in the COVID-19 diagnostic panel described by the US Centers for Disease Control and Prevention (CDC, https://www.cdc.gov/).

### Melting curves (in the intercalating dye analysis)

In the dye experiments, shinyCurves allows users to plot melting curves to exclude from the final calling those samples lacking reliable and unique melting temperature (Tm) peaks. These plots are generated using the *meltcurve* function from the ‘qpcR’ package in R [13]. As a result, users can download a table including only those plate wells that contain samples with unique Tm peaks that meet the established criteria, together with the Tm value and plot assigned to each well. This table must be included in the calling analysis as a prior filtering step (*ID_well* file).

### Calling analysis

After uploading the input data, users are allowed to fine-tune a series of parameters that will be used to classify samples as Positive, Negative or Undetermined. In general, two types of calling criteria are allowed, namely, the Ct value of the analyzed viral gene(s) (compulsory) and the estimated viral RNA copy number (optional). A human endogenous control is included to make sure that the nucleic acid extraction worked. Adjustable parameters include, among others: use of a viral DNA standard curve (yes/no), use of duplicates (yes/no) and number of “positive” viral genes to consider a sample Positive. Calling results are presented in a downloadable table. For more details on the calling criteria, see Additional file 3, where the algorithm of the full Calling Analysis has been described in detail.

### Amplification curves (in the fluorescent probe analysis)

After performing the calling analysis in probe experiments, shinyCurves allows to plot General Amplification Curves including all the samples, as well as individual Amplification Curves of samples classified as Undetermined. Each Undetermined sample is plotted independently, together with those with a final calling. Upon visual inspection of Undetermined sample curves, the user can decide whether these fit a sigmoidal distribution, and therefore represent a specific amplification, or not.

## Conclusions

To our knowledge, this is the first tool designed to automatize the calling of clinical samples containing pathogen nucleic acids through qRT-PCR, and that is completely flexible as regards the user’s requirements and experimental settings. In fact, several open-access software packages and tools for the analysis of qPCR data already exist (see review by [14]). However, some of them either have been discontinued (CopyCaller) or are no longer maintained [15] or need a subscription or license (Cy0 Method, https://www.cy0method.org/, [16]). Moreover, the alternatives for drawing melting curves other than proprietary software are very limited. As far as we know, the qpcR R package is the only available free package, but no graphical user interface is provided [17].

In summary, shinyCurves is a user-friendly application that analyzes and allows the visualization of qRT-PCR data coming from different amplification methods and platforms. It is easily accessible for any user profile, as no programming skills are required. shinyCurves is set up automatically in the shinyapps.io server, making basic Internet connection its only requirement. Its minimal requirements make it a ready-to-use tool applicable to the clinical routine. Therefore, we conclude that it is a significant improvement in analytical capacity, speed and reproducibility, which are key factors in pathogen detection analyses, especially in COVID-19 times.

## Supplementary Information

**Additional file 1.** COVID-19 toy dataset. A COVID-19 toy dataset containing example files coming from (a) a fluorescent probe analysis (Applied Biosystems Quant Studio) and (b) an intercalating dye analysis (BioRad CFX).

**Additional file 2.** shinyCurves manual. Contains a precise description of all the options implemented in shinyCurves, as well as a tutorial describing the analysis of the two COVID-19 datasets provided.

**Additional file 3.** Result assignment criteria in the Calling Analysis. In the upper section, samples are assigned a result based on their viral gene Ct values (compulsory). First, when duplicates are included in the analysis, mean Ct is calculated and samples with divergent duplicates are marked as Undetermined. Then, in (2), mean (samples with duplicates) or individual (samples with no duplicates) Ct values are compared against the maximum Ct value (MaxCt) selected by the user and each sample is assigned a result (Positive, Negative, Check Copy Number). In (3), the total number of Positive, Negative or Check Copy Number assignments is compared to the number of Positive genes necessary to assign a sample as Positive (inputted by the user) and the sample is assigned a final result (Positive, Negative, Undetermined or Check Copy Number). If the user does not want to consider the copy number as a result assignation criterion, the analysis is over. Otherwise, the analysis continues in the blue section in which samples are assigned a result based on their estimated viral DNA copy number (optional). In (4a), the estimated copy number is compared to the minimum copy number inputted by the user and the gene is assigned as Positive or Undetermined. Finally, in (4b), similarly to (3), the gene result assignment is compared to the number of Positive genes necessary to assign a sample as Positive and the sample is assigned as either Positive or Undetermined.

## Data Availability

Project name: shinyCurves. Project home page: https://biosol.shinyapps.io/shinycurves/. Operating system(s): Platform independent. Programming language: R. Other requirements: basic Internet connection. License: CC BY-NC-SA 4.0. Any restrictions to use by non-academics: CC BY-NC-SA 4.0. The toy dataset analyzed in this study can be downloaded from https://biosol.shinyapps.io/shinycurves/.

## Abbreviations

qRT-PCR: reverse transcription polymerase chain reaction
WHO: World Health Organization
FDA: Food and Drug Agency
coBIG: COVID-19 Basque Inter-Institutional Group
CDC: US Centers for Disease Control and Prevention
Tm: melting temperature
