# Supplementary figures and images for: shinyCurves, a shiny web application to analyse multisource qPCR amplification data: a COVID-19 case study
Source: BMC Bioinformatics. 2021 Oct 3;22:476. doi: 10.1186/s12859-021-04392-1 (PMC8487674; doi:10.1186/s12859-021-04392-1)

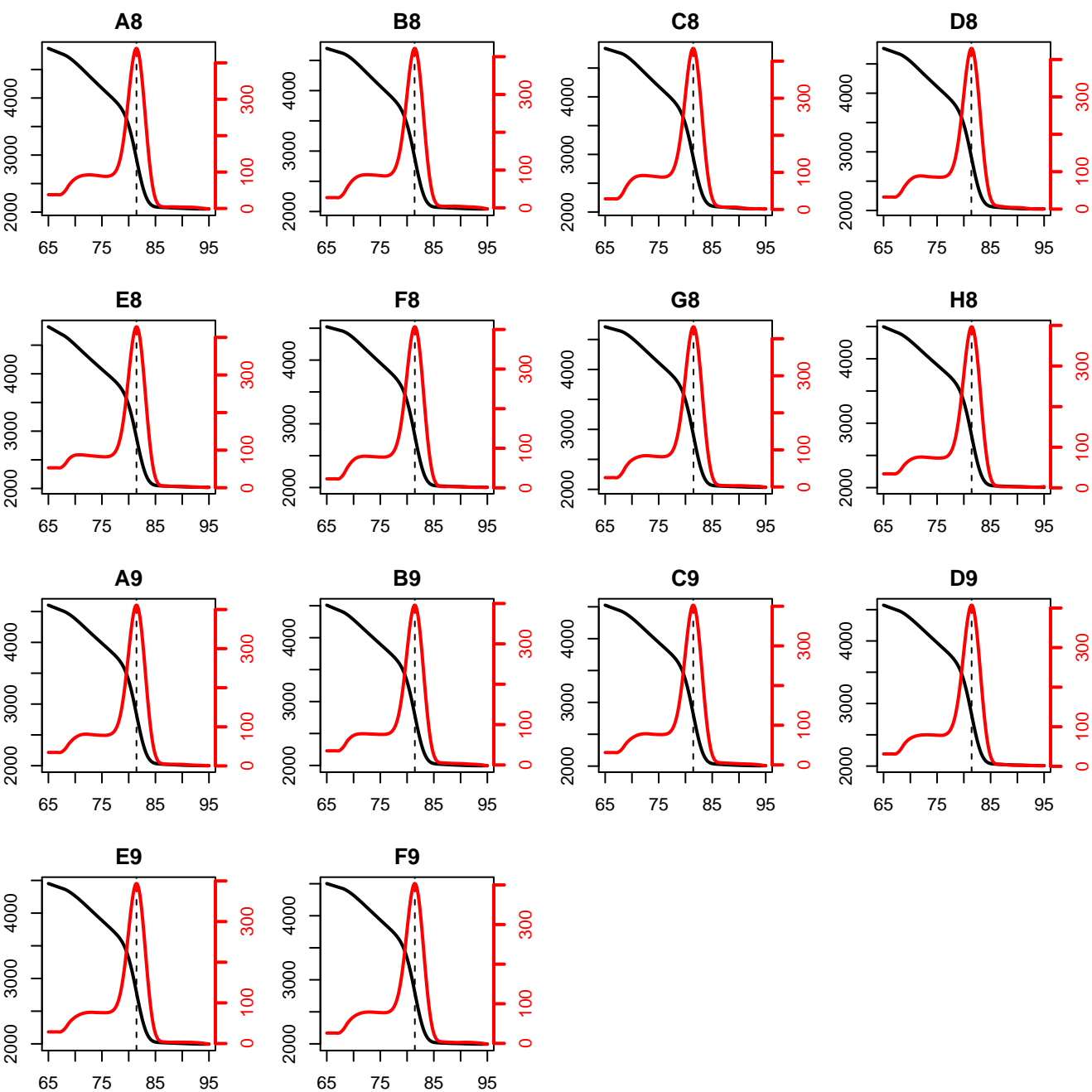

Supplement: Supplementary file 1 — Additional file 1. COVID-19 toy dataset. A COVID-19 toy dataset containing example files coming from (a) a fluorescent probe analysis (Applied Biosystems Quant Studio) and (b) an intercalating dye analysis (BioRad CFX). [file 12859_2021_4392_MOESM1_ESM.zip › toy/DYE-Biorad/output/MeltingCurves_H30.pdf]

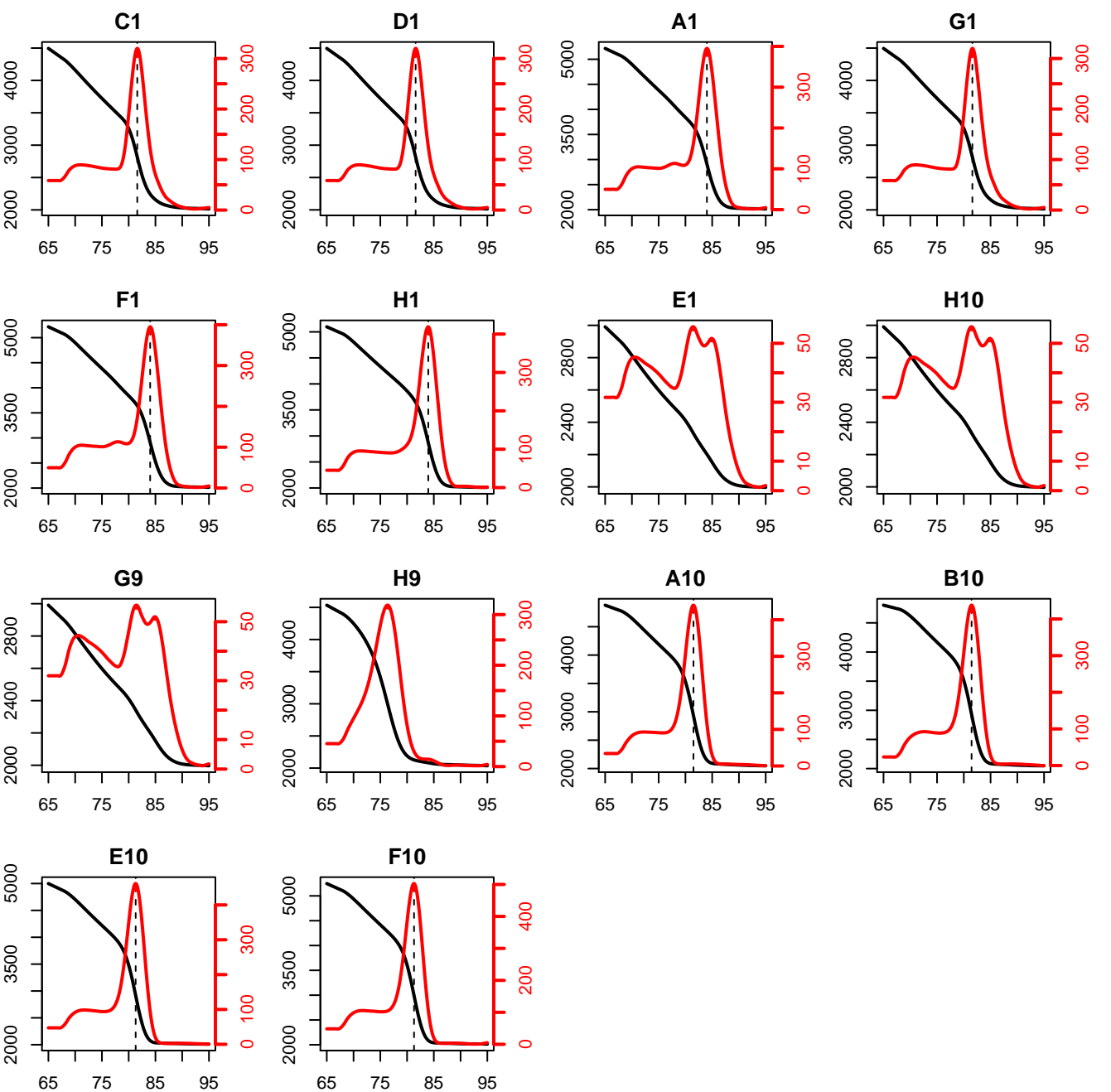

Supplement: Supplementary file 1 — Additional file 1. COVID-19 toy dataset. A COVID-19 toy dataset containing example files coming from (a) a fluorescent probe analysis (Applied Biosystems Quant Studio) and (b) an intercalating dye analysis (BioRad CFX). [file 12859_2021_4392_MOESM1_ESM.zip › toy/DYE-Biorad/output/MeltingCurves_N.pdf]

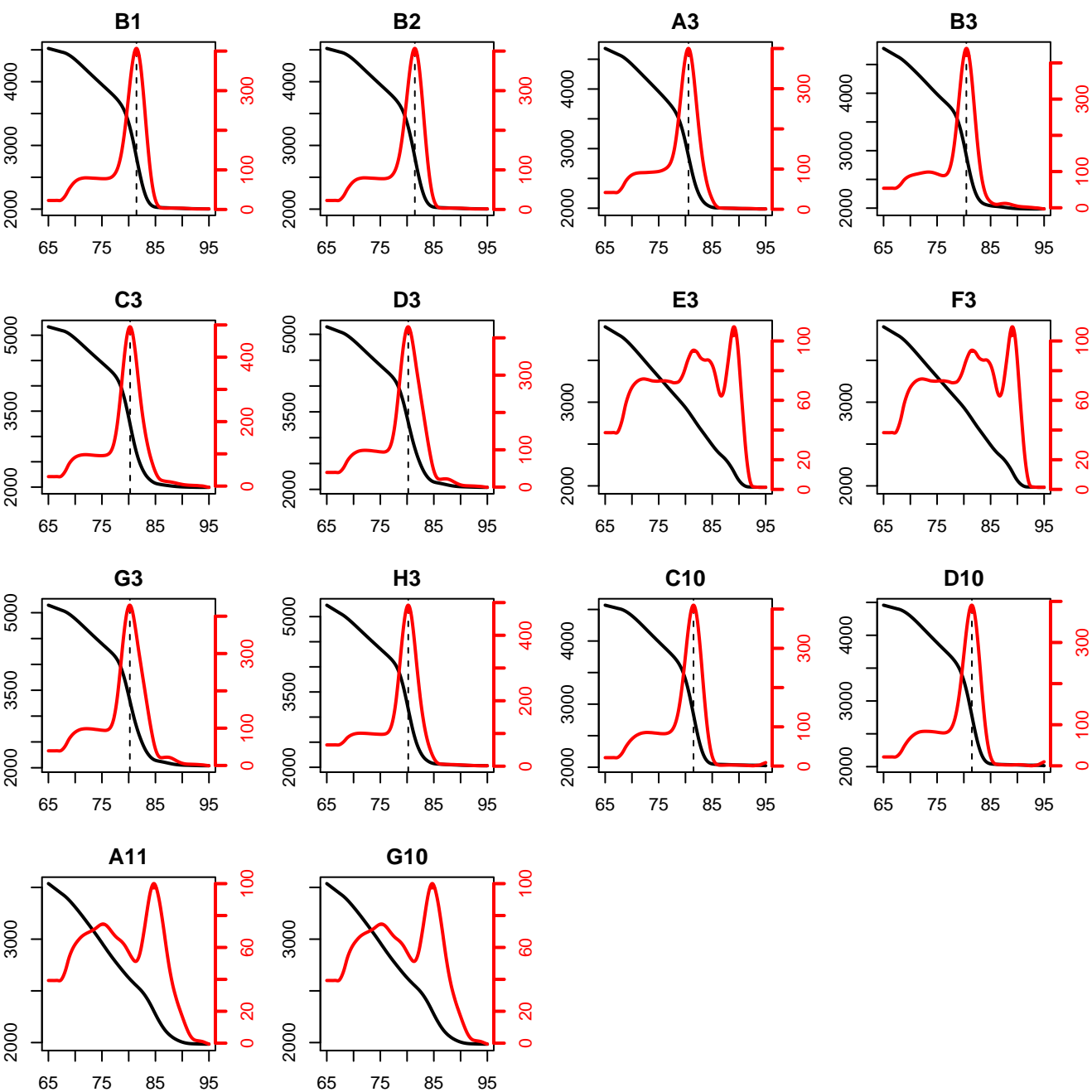

Supplement: Supplementary file 1 — Additional file 1. COVID-19 toy dataset. A COVID-19 toy dataset containing example files coming from (a) a fluorescent probe analysis (Applied Biosystems Quant Studio) and (b) an intercalating dye analysis (BioRad CFX). [file 12859_2021_4392_MOESM1_ESM.zip › toy/DYE-Biorad/output/MeltingCurves_S.pdf]

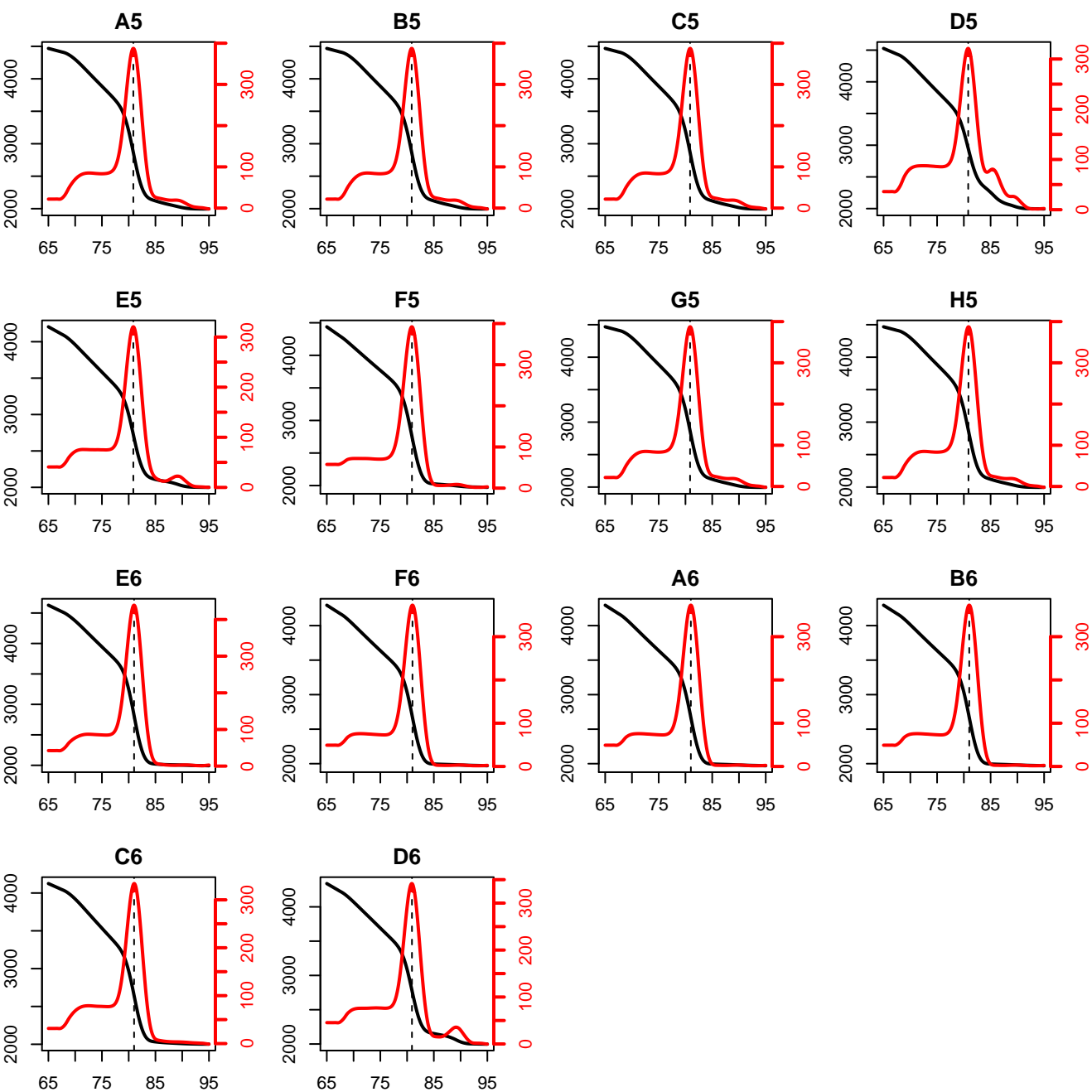

Supplement: Supplementary file 1 — Additional file 1. COVID-19 toy dataset. A COVID-19 toy dataset containing example files coming from (a) a fluorescent probe analysis (Applied Biosystems Quant Studio) and (b) an intercalating dye analysis (BioRad CFX). [file 12859_2021_4392_MOESM1_ESM.zip › toy/DYE-Biorad/output/MeltingCurves_RdRp.pdf]

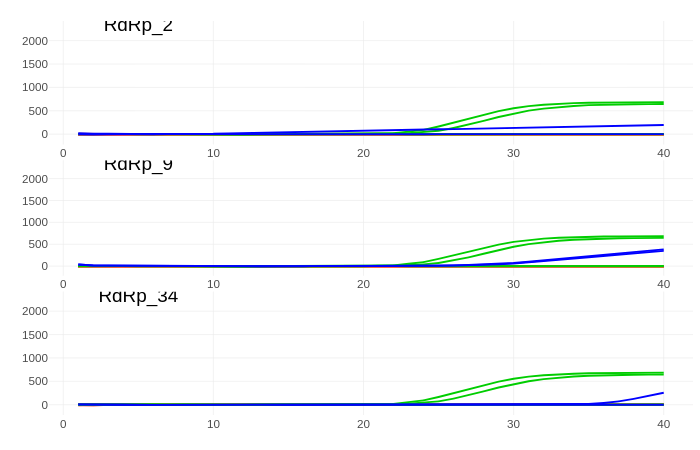

Supplement: Supplementary file 1 — Additional file 1. COVID-19 toy dataset. A COVID-19 toy dataset containing example files coming from (a) a fluorescent probe analysis (Applied Biosystems Quant Studio) and (b) an intercalating dye analysis (BioRad CFX). [file 12859_2021_4392_MOESM1_ESM.zip › toy/PROBE-Applied/output/RdRp_IndetAmpCurves.png]

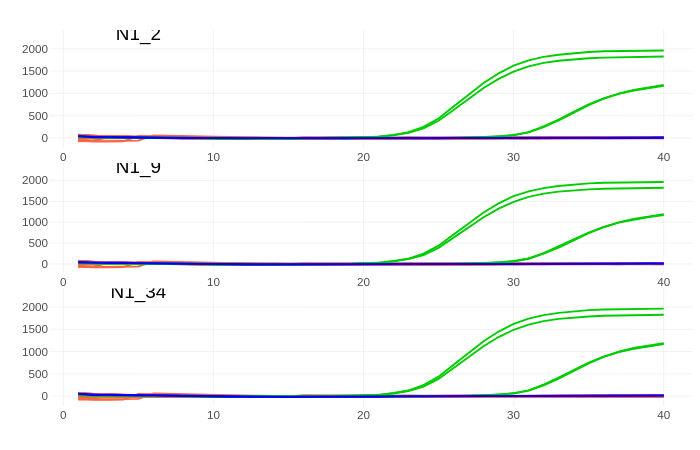

Supplement: Supplementary file 1 — Additional file 1. COVID-19 toy dataset. A COVID-19 toy dataset containing example files coming from (a) a fluorescent probe analysis (Applied Biosystems Quant Studio) and (b) an intercalating dye analysis (BioRad CFX). [file 12859_2021_4392_MOESM1_ESM.zip › toy/PROBE-Applied/output/N1_IndetAmpCurves.png]

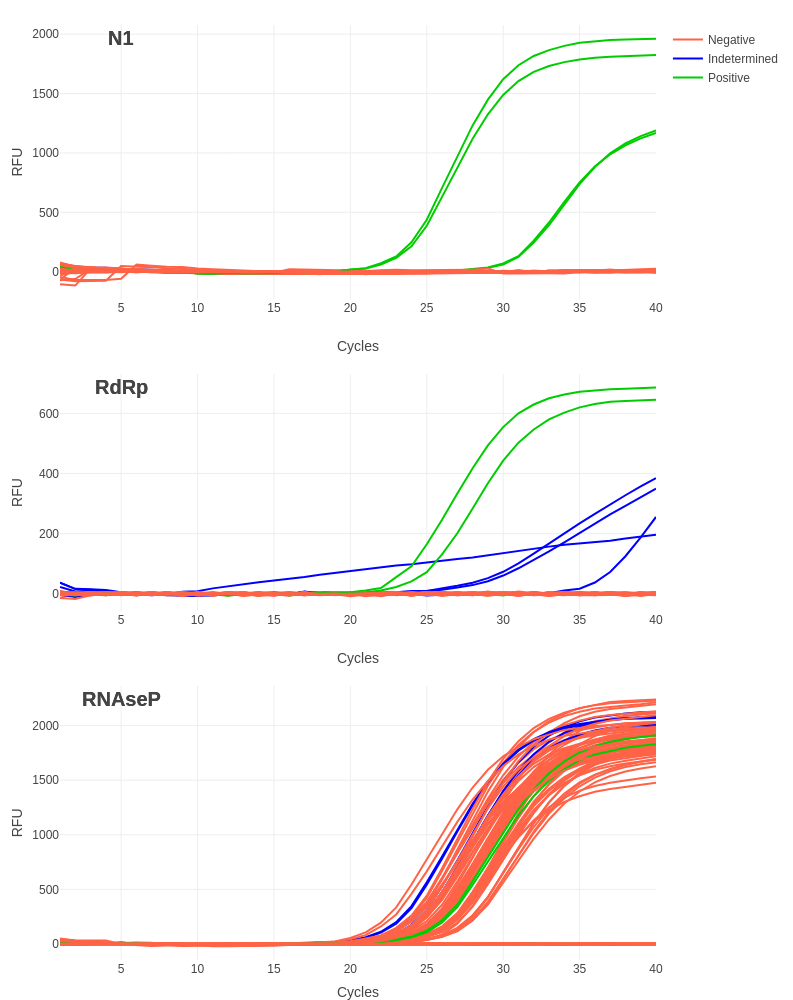

Supplement: Supplementary file 1 — Additional file 1. COVID-19 toy dataset. A COVID-19 toy dataset containing example files coming from (a) a fluorescent probe analysis (Applied Biosystems Quant Studio) and (b) an intercalating dye analysis (BioRad CFX). [file 12859_2021_4392_MOESM1_ESM.zip › toy/PROBE-Applied/output/GeneralAmplificationCurves.png]

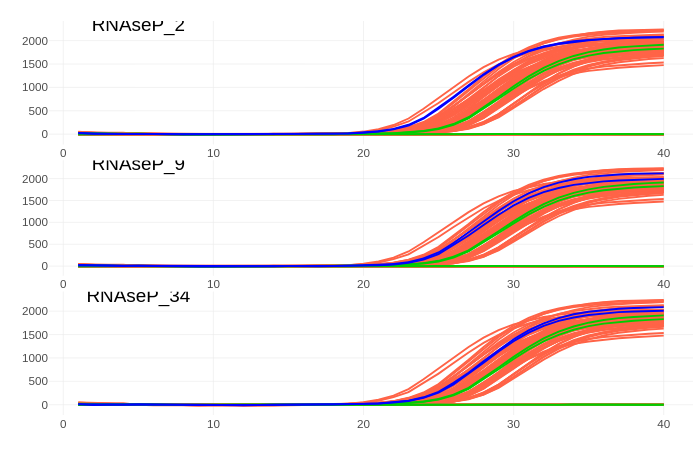

Supplement: Supplementary file 1 — Additional file 1. COVID-19 toy dataset. A COVID-19 toy dataset containing example files coming from (a) a fluorescent probe analysis (Applied Biosystems Quant Studio) and (b) an intercalating dye analysis (BioRad CFX). [file 12859_2021_4392_MOESM1_ESM.zip › toy/PROBE-Applied/output/RNAseP_IndetAmpCurves.png]

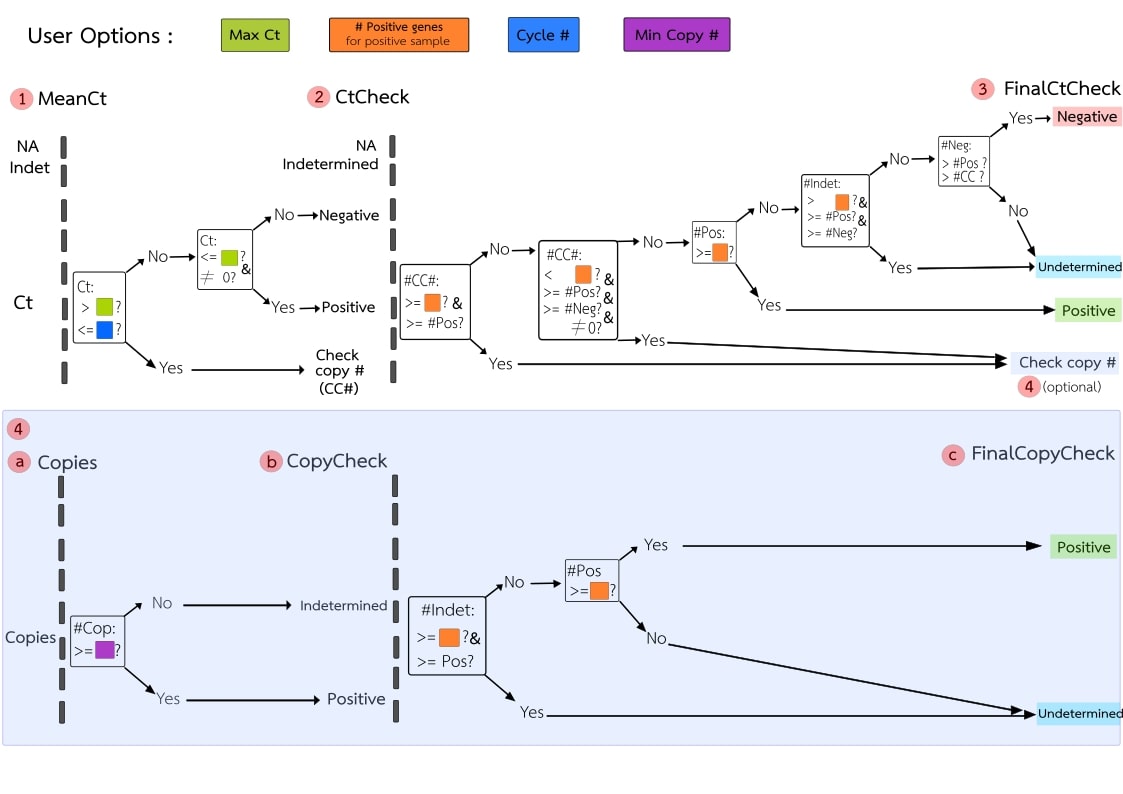

Supplement: Supplementary file 3 — Additional file 3. Result assignment criteria in the Calling Analysis. In the upper section, samples are assigned a result based on their viral gene Ct values (compulsory). First, when duplicates are included in the analysis, mean Ct is calculated and samples with divergent duplicates are marked as Undetermined. Then, in (2), mean (samples with duplicates) or individual (samples with no duplicates) Ct values are compared against the maximum Ct value (MaxCt) selected by the user and each sample is assigned a result (Positive, Negative, Check Copy Number). In (3), the total number of Positive, Negative or Check Copy Number assignments is compared to the number of Positive genes necessary to assign a sample as Positive (inputted by the user) and the sample is assigned a final result (Positive, Negative, Undetermined or Check Copy Number). If the user does not want to consider the copy number as a result assignation criterion, the analysis is over. Otherwise, the analysis continues in the blue section in which samples are assigned a result based on their estimated viral DNA copy number (optional). In (4a), the estimated copy number is compared to the minimum copy number inputted by the user and the gene is assigned as Positive or Undetermined. Finally, in (4b), similarly to (3), the gene result assignment is compared to the number of Positive genes necessary to assign a sample as Positive and the sample is assigned as either Positive or Undetermined. [file 12859_2021_4392_MOESM3_ESM.jpg]
